# Effect of Implementing an Informatization Case Management Model on the Management of Chronic Respiratory Diseases in a General Hospital: Retrospective Controlled Study

**DOI:** 10.2196/49978

**Published:** 2024-06-19

**Authors:** Yi-Zhen Xiao, Xiao-Jia Chen, Xiao-Ling Sun, Huan Chen, Yu-Xia Luo, Yuan Chen, Ye-Mei Liang

**Affiliations:** 1 Department of Pulmonary and Critical Care Medicine, Yulin First People’s Hospital, Yulin, China; 2 Department of Nursing, Yulin First People’s Hospital, Yulin, China

**Keywords:** chronic disease management, chronic respiratory disease, hospital information system, informatization, information system, respiratory, pulmonary, breathing, implementation, care management, disease management, chronic obstructive pulmonary disease, case management

## Abstract

**Background:**

The use of chronic disease information systems in hospitals and communities plays a significant role in disease prevention, control, and monitoring. However, there are several limitations to these systems, including that the platforms are generally isolated, the patient health information and medical resources are not effectively integrated, and the “Internet Plus Healthcare” technology model is not implemented throughout the patient consultation process.

**Objective:**

The aim of this study was to evaluate the efficiency of the application of a hospital case management information system in a general hospital in the context of chronic respiratory diseases as a model case.

**Methods:**

A chronic disease management information system was developed for use in general hospitals based on internet technology, a chronic disease case management model, and an overall quality management model. Using this system, the case managers provided sophisticated inpatient, outpatient, and home medical services for patients with chronic respiratory diseases. Chronic respiratory disease case management quality indicators (number of managed cases, number of patients accepting routine follow-up services, follow-up visit rate, pulmonary function test rate, admission rate for acute exacerbations, chronic respiratory diseases knowledge awareness rate, and patient satisfaction) were evaluated before (2019‐2020) and after (2021‐2022) implementation of the chronic disease management information system.

**Results:**

Before implementation of the chronic disease management information system, 1808 cases were managed in the general hospital, and an average of 603 (SD 137) people were provided with routine follow-up services. After use of the information system, 5868 cases were managed and 2056 (SD 211) patients were routinely followed-up, representing a significant increase of 3.2 and 3.4 times the respective values before use (*U*=342.779; *P*<.001). With respect to the quality of case management, compared to the indicators measured before use, the achievement rate of follow-up examination increased by 50.2%, the achievement rate of the pulmonary function test increased by 26.2%, the awareness rate of chronic respiratory disease knowledge increased by 20.1%, the retention rate increased by 16.3%, and the patient satisfaction rate increased by 9.6% (all *P*<.001), while the admission rate of acute exacerbation decreased by 42.4% *(P*<.001) after use of the chronic disease management information system.

**Conclusions:**

Use of a chronic disease management information system improves the quality of chronic respiratory disease case management and reduces the admission rate of patients owing to acute exacerbations of their diseases.

## Introduction

Chronic obstructive pulmonary disease (COPD) and asthma are examples of common chronic respiratory diseases. The prevalence of COPD among people 40 years and older in China is estimated to be 13.7%, with the total number of patients reaching nearly 100 million. The lengthy disease cycle, recurrent acute exacerbations, and low control rate were found to have a significant impact on the prognosis and quality of life of middle-aged and older patients with COPD [1,2]. Therefore, to decrease the morbidity and disability rates and enhance the quality of life of all patients with chronic respiratory diseases, it is crucial to investigate effective prevention and treatment methods and establish a life cycle management model for chronic respiratory diseases.

Since the development of information technology, the internet and medical technology have been applied to the management of chronic diseases [3]. The chronic disease information systems adopted in hospitals and communities, along with mobile medical apps, can enhance the self-management capabilities of patients and play a significant role in disease prevention, control, and monitoring [4-9]. However, the existing platforms are generally isolated, the patient health information and medical resources are not effectively integrated, and the Internet Plus Healthcare technology model is not implemented throughout the patient consultation process [3,9].

Yulin First People’s Hospital developed a chronic disease management information system based on the hospital information system (HIS) to fully and effectively utilize the medical resources in hospitals and to better support and adapt the system to the needs of patients with chronic diseases. In this study, we evaluated the impact of the use of this system on the efficacy of case management for patients with chronic respiratory diseases.

## Methods

### Chronic Respiratory Diseases Case Management Model Prior to Implementation of the Chronic Disease Management Information System

Yulin First People’s Hospital is a public grade-3 general hospital with 2460 open beds, a specialty clinic in the Department of Pulmonary and Critical Care Medicine, and 180 beds in the Inpatient Department. Chronic respiratory diseases case management was initiated in 2019, which did not involve the use of an information system and was implemented by a chronic respiratory diseases case management team led by two nurses qualified as case managers, one chief physician, two supervisor nurses, and one technician. Under this system, patients with COPD, bronchial asthma, bronchiectasis, pulmonary thromboembolism, lung cancer, and lung nodules were managed using the traditional inpatient-outpatient-home chronic respiratory diseases case management model, including 1024 cases managed from 2019 to 2020. Except for medical prescriptions and electronic medical records, the patient case management information such as the basic information form, follow-up form, patient enrollment form, inpatient follow-up register, patient medication and inhalation device use records, smoking cessation and vaccination records, and pulmonary rehabilitation and health education records was managed using Microsoft Excel forms that were regularly printed for filing.

### Establishment of a Management Information System for Chronic Diseases

The information carrier forming the basis of the management information system is constituted by the model of internet technology, chronic disease case management models, and overall quality management. The key technology is to establish a scientific, refined, and feasible follow-up pathway according to the methods and procedures of chronic disease case management based on the guidelines for the diagnosis and treatment of single chronic diseases. The closed-loop management of the clinical pathway was conducted in accordance with the Deming cycle (plan-do-check-act), and dynamic monitoring of single-disease health-sensitive and quality-sensitive indicators was carried out. The successfully developed system was installed on the hospital server to connect personal terminals (medical terminals and customer apps) to the existing HIS, which includes electronic medical records and medical advice.

Using the single-disease path assessment or plan scale as a framework, the system can automatically collect and integrate the majority of the medical information of patients with chronic respiratory diseases and provide these patients with inpatient, outpatient, and home intelligent medical services. Patients with chronic diseases who enroll in use of the system can use the app to schedule appointments for medical guidance, payment, and result queries; receive health guidance information; perform self-health assessments; write a treatment diary; and obtain medical communication materials.

**Figure 1.**
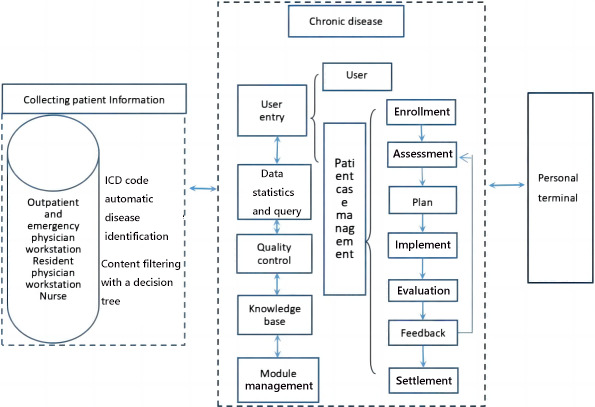
Main structure of the hospital information system–based chronic disease management information system. *ICD*: *International Classification of Diseases*.

The medical terminal consists of five functional modules: user entry, data statistics and query, quality control, knowledge base, and module management. As the core of the system, the user entry module can manage case information in seven steps: enrollment, assessment, planning, implementation, feedback, evaluation, and settlement [10-14]. Each step has a corresponding assessment record scale as well as the health-sensitive and quality-sensitive indicators. The structure of the HIS-based chronic disease management information system is shown in Figure 1.

### Implementation of the Chronic Disease Information Management System

#### Overview

Using the chronic disease management information system, two full-time case managers oversaw the case management of 2747 patients diagnosed with six diseases among chronic respiratory diseases between 2021 and 2022. The operation process was broken down into enrollment, assessment, planning, implementation, evaluation, feedback, and settlement stages.

#### Enrollment

Case managers entered the system through the medical app, selected a disease and an enrolled patient from the list of patients (the system automatically captures the patient’s name and ID number according to the *International Classification of Diseases* [*ICD*] code) in accordance with the chronic respiratory diseases diagnostic criteria to sign the enrollment contract and determine the relationship between the personal information and data [15-19].

#### Assessment

The system can be seamlessly integrated with multiple workstations on the HIS to automatically capture the basic information, electronic medical records, medical advice, and inspection materials, and can generate questionnaires or assessment scales for patients with chronic respiratory diseases such as the COPD Assessment Test, Asthma Control Test, modified Medical Research Council scale, form for lung function test results, inhalation device technique evaluation form, 6-minute walk test record, rehabilitation assessment form, health promotion form, and nutritional assessment form. The above materials can be added or removed based on the requirements for individual patients.

#### Planning

The case managers drafted the follow-up plan based on the patient assessment criteria and included the patients on the 1-, 3-, 6-, and 12-month follow-up lists. If the patient satisfied the self-management and indicator control requirements after follow-up, they could be settled and included in the annual follow-up cohort. Case managers can set up follow-up warning and treatment, involving the return visit plan, health education, follow-up content, pathway, and time, and notify the patients and nurses on day 7 and at months 1, 3, 6, and 12 after discharge. The nurses should promptly deal with patients who miss their scheduled follow-up visit.

#### Implementation

During the inpatient or outpatient care, supervising physicians, nurses, and patients collaborated with each other to implement the treatment. Case managers monitored the patients, evaluated them, documented the results, interpreted various test indicators, and provided health guidance. The chronic disease management information system acquired the corresponding data for chronic disease–sensitive indicators from outpatient and inpatient orders and medical records automatically. The chronic respiratory diseases management team reviewed the patients’ conditions and the dynamics of chronic disease–sensitive indicators to make accurate decisions based on the current situation. The outpatient physicians obtained the single-disease package advice and personalized prescriptions to modify the diagnosis and treatment scheme.

#### Evaluation

Case managers highlighted evaluation and health education. First, they assessed and examined the content of the previous education and recorded and analyzed the patients’ conditions, medication, diet, nutrition, rehabilitation exercises, and self-management. Second, they prepared the personalized health education plan, return visit plan, and rehabilitation plan, and used standardized courseware, educational videos, and health prescriptions to provide the patients with one-on-one health guidance. Finally, they sent the management tasks and educational contents to the phones of the patients for consolidating the learning in the hospital, as an outpatient, and at home.

#### Feedback

Patients can access their biochemical, physical, and chemical data as well as chronic disease–sensitive indicators in the hospital, as an outpatient, and at home for self-health management. Case managers can also perform online assessment, appraisal, and guidance via telephone, WeChat, and the chronic disease information system and record the data. Client mobile terminals can receive SMS text message alerts and the main interface of the chronic disease information system would display reminders of follow-up and return visits within ±7 days.

#### Settlement

If a patient was out of contact for 3 months, died, or refused to accept the treatment, case managers could settle the case.

### Evaluation of the Effect of Implementing the Chronic Disease Management Information System

#### Evaluation Method

In accordance with case quality management indicators [20], two full-time case managers collected and evaluated data in the process of the follow-up procedure. To reduce the potential for evaluation bias, the case managers consistently communicated and learned to standardize the evaluation method. The cases were divided based on different chronic respiratory diseases case management models (ie, before and after use of the chronic disease information system). The following case management quality indicators were evaluated under the noninformation system management model (2019‐2020) and under the chronic disease management information system model (2021‐2022): number of managed cases, number of patients accepting routine follow-up services, follow-up visit rate, pulmonary function test rate, admission rate for acute exacerbations, chronic respiratory diseases knowledge awareness rate, and patient satisfaction. Excel sheets were used to acquire data prior to incorporation of the chronic disease management information system into the new information system.

#### Evaluation Indicators

The annual number of cases was calculated as the sum of the number of newly enrolled patients and the number of initially enrolled patients. The number of cases was calculated as the sum of the number of cases in different years. The number of routine follow-up visits represents the number of patients who completed the treatment plan. The follow-up visit rate was calculated as the number of completed follow-up visits in the year divided by the number of planned follow-up visits in the same year. The pulmonary function test rate was calculated as the number of pulmonary function tests completed for patients scheduled for follow-up during the year divided by the number of pulmonary function tests for patients scheduled for follow-up during the year. The admission rate for acute exacerbations was calculated as the number of recorded patients admitted to the hospital due to acute exacerbations divided by the total number of patients recorded. The chronic respiratory diseases knowledge awareness rate was determined by the number of people having sufficient knowledge divided by the total number of people tested. This knowledge indicator was based on the self-prepared chronic respiratory diseases knowledge test scale, which consists of 10 items determined using the Delphi method (following expert consultation) through review of the literature, including common symptoms, disease hazards, treatment medication, diet, living habits, exercise, negative habits affecting the disease, regular review items, effective methods for cough and sputum removal, appointments, and follow-ups. The content of the questionnaire was refined by disease type, and the reviewers included 11 personnel with the title of Deputy Chief Nurse or above in the Internal Medicine Department of the hospital. The expert authority coefficients were 0.85 and 0.87 and the coordination coefficients were 0.50 and 0.67 for the two rounds of review, respectively; the *χ*^2^ test showed a statistically significant value of *P*=.01. Patient satisfaction was assessed with a self-made questionnaire that showed good internal reliability (Cronbach α=0.78) and content validity (0.86). The questionnaire items included the reminder of return visits, practicability of health education content, and service attitude of medical staff; the full-time case managers surveyed the patients (or their caregivers) at the time of return visits after the third quarter of each year. Satisfaction items were rated using a 5-point Likert scale with a score of 1‐5, and a mean ≥4 points for an individual indicated satisfaction. Patient satisfaction was then calculated as the number of satisfied patients divided by the total number of managed patients.

### Statistical Analysis

SPSS 16.0 software was used for data analysis. The Mann-Whitney *U* test was performed to compare continuous variables between groups and the *χ*^2^ test was performed to compare categorical variables between groups. *P*<.05 indicated that the difference was statistically significant.

### Ethical Considerations

The study was conducted in accordance with the principles of the Declaration of Helsinki. This study received approval from the Ethics Committee of Yulin First People’s Hospital (approval number: YLSY-IRB-RP-2024005). The study did not interfere with routine diagnosis and treatment, did not affect patients’ medical rights, and did not pose any additional risks to patients. Therefore, after discussion with the Ethics Committee of Yulin First People’s Hospital, it was decided to waive the requirement for informed consent from patients. Patients’ personal privacy and data confidentiality have been upheld throughout the study.

## Results

### Characteristics of Patient Populations Before and After Implementation of the Information System

There was no significant difference in age and gender distributions in the patient populations that received care before and after implementation of the chronic disease management information system (Table 1).

**Table 1.** General characteristics of the patient populations under case management before and after use of the chronic disease management information system.

Characteristic		Before use (n=1024)	After use (n=2747)	*χ*² value	*df*	*P* value
**Gender, n (%)**				1.046	1	.31
	Men	677 (66.1)	1767 (64.3)			
	Women	347 (33.9)	980 (35.7)			
**Age group (years), n (%)**				0.997	3	.80
	<30	26 (2.6)	73 (2.7)			
	30-59	370 (36.1)	1013 (36.9)			
	60-79	510 (49.8)	118 (11.5)			
	>80	1322 (48.1)	339 (12.3)			

### Comparison of Workload Before and After Implementation of the Information Management System

Before use of the system, 1808 cases were managed, with a mean of 603 (SD 137) cases having routine follow-up visits. After use of the system, 5868 cases were managed, with a mean of 2056 (SD 211) routine follow-up visits. Therefore, the number of managed cases and the number of follow-up visits significantly increased by 3.2 and 3.4 times, respectively, after use of the system *(U*=342.779; *P<*.001).

### Comparison of Quality Indicators of Managed Cases Before and After Implementation of the Information System

The quality indicators in the two groups are summarized in Table 2. Compared with the corresponding indicators before use of the system, the follow-up visit rate increased by 50.2%, the pulmonary function test rate increased by 26.2%, the chronic respiratory diseases knowledge awareness rate increased by 20.1%, the retention rate increased by 16.3%, and the patient satisfaction increased by 9.6%; moreover, the admission rate for acute exacerbations decreased by 42.4%.

**Table 2.** Comparison of case management quality indicators before and after implementation of the chronic diseases information management system.

Quality indicators	Before use (n=1024), n (%)	After use (n=2747), n (%)	*χ*² value (*df*=1)	*P* value
Subsequent visit rate	209 (20.4)	1939 (70.6)	7.660	<.001
Lung function test achievement rate	190 (18.6)	1231 (44.8)	2.190	<.001
CRD^a^ knowledge awareness rate	443 (43.3)	1742 (63.4)	1.243	<.001
Retention rate	787 (76.9)	2560 (93.2)	1.995	<.001
Acute exacerbation admission rate	663 (64.7)	613 (22.3)	5.999	<.001
Patient satisfaction	862 (84.2)	2577 (93.8)	86.190	.01

a CRD: chronic respiratory disease.

## Discussion

### Principal Findings

The main purpose of this study was to build a chronic disease management information system and apply it to the case management of chronic respiratory diseases. Our evaluation showed that the chronic disease management information system not only improves the efficiency and quality of case management but also has a benefit for maintaining the stability of the condition for patients with respiratory diseases, reduces the number of acute disease exacerbations, increases the rate of outpatient return, and improves patients’ adherence with disease self-management. Thus, a chronic disease management information system is worth popularizing and applying widely.

### Value of the HIS-Based Chronic Disease Management Information System

Chronic diseases constitute a significant public health issue in China. Public hospitals play important roles in the health service system, particularly large-scale public hospitals with the most advanced technologies, equipment, and enormous medical human resources, which can greatly aid in the diagnosis and treatment of diseases and also serve as important hubs for the graded treatment of chronic diseases. Moreover, a significant number of patients with chronic diseases visit large hospitals, making them important sources of big data on chronic diseases [21]. Adoption of an HIS-based chronic disease management information system can make full use of and exert the advantages of large-scale public hospitals in terms of labor, technology, and equipment in the diagnosis, treatment, and prevention of chronic diseases; enhance the cohesiveness of the case management team in chronic disease management; and achieve prehospital, in-hospital, and posthospital continuity of care for patients with chronic diseases. Overall, use of a chronic disease management information system can enhance the quality and efficiency of chronic disease management and lay a good foundation for teaching and research on chronic diseases.

### Improved Efficiency of Case Management

China was relatively late in applying case management practices, and chronic disease management has traditionally been primarily conducted offline [14,20] or supplemented by management with apps and WeChat [7,8]. Traditional case management methods require case managers to manually search, record, store, query, count, and analyze information. This manual process necessitates substantial time and makes it challenging to realize a comprehensive, systematic, and dynamic understanding of patient information, resulting in a small number of managed cases and follow-up visits. With the application of information technology, use of an HIS-based chronic disease monitoring and case management system can automatically extract and integrate patient information, thereby increasing the efficiency of chronic disease management and reduce costs [4,22]. In this study, two case managers played leading roles both before and after implementation of the information system; however, compared with the situation before the use of the system, the numbers of both managed cases and of follow-up visits increased, reaching 3.2 and 3.4 times the preimplementation values, respectively. The information system can automatically obtain a patient’s name and ID number based on the *ICD* code, which can expand the range of enrollment screening and appoint the register of patients as planned. In addition, the information system can automatically obtain outpatient, inpatient, and home medical information for the postillness life cycle management of patients. Moreover, the intuitive, clear, and dynamic indicator charts on the system can save a significant amount of time for diagnosis and treatment by medical staff, while the paperless office and online data-sharing functions can essentially solve the problem of managing files by case managers to ultimately enhance efficiency.

### Improved Quality of Case Management

According to evidence-based medicine, the seven steps of case management represent the optimal clinical pathway [10-14,22]. In this study, the concept of an Internet-Plus medical service was introduced; that is, the chronic disease management information system was established based on the HIS data and case management model [22] and the function of a mobile medical terminal app was incorporated in the system [6,7]. Compared with the noninformation system case management model, this system has several advantages. First, owing to the swift management mode, it can overcome the limitations of time and space [4-8]. Second, multichannel health education and communication can enhance patients’ knowledge and skills, as well as their compliance with self-management, based on diversified forms of image data such as graphics and audio [6,22]. Third, the use of intelligent management can remind doctors and patients to complete management work and follow-up visits as planned, and to perform intelligent pushes of patient outcome indicators to improve confidence in the treatment [22]. Fourth, this system enables information sharing and big data analysis, as well as multidisciplinary diagnosis and treatment based on the matching of doctor-patient responsibility management, which can be more conducive to the precise health management of patients.

Compared with the traditional case management model, information-based case management significantly increased the follow-up visit rate, lung function test rate, chronic respiratory diseases knowledge awareness rate of patients, patient satisfaction rate, and retention rate. Among these indicators, the follow-up visit rate and lung function test rate represent aspects related to the patients’ own management of their condition [1]. The results of this study are consistent with previous findings related to information-based management of chronic diseases in China, demonstrating that such a management system was more conducive to planned, systematic, and personalized education and follow-up by the case management team, thereby promoting the virtuous cycle of compliance with self-management and reducing the number of acute exacerbations among patients with chronic respiratory diseases, ultimately enhancing the precision of medical resource allocation and hospital management [22,23].

### Helping Patients With COPD Maintain Stability of Their Condition

The admission rate for acute exacerbations serves as a common indicator of the quality of the treatment of chronic respiratory diseases [23]. The deployment of a clinical pathway–based hospital case management information system significantly reduced the admission rate for acute exacerbations and enhanced the quality of treatment for chronic respiratory diseases, indicating its high clinical significance. There are several reasons for these observed benefits. First, home care and self-management are essential in the management of chronic respiratory diseases. The information-based case management model improved the patients’ knowledge and skills along with their compliance with self-management. Consequently, the standardized self-management process helped to reduce the number of acute exacerbations of chronic respiratory diseases and thus lowered the admission rate. Second, the information-based case management model increased the regular return rate, which allowed the medical staff to identify the potential risk factors for acute exacerbations in a timely manner, deal with them when they occur, and prepare personalized treatment plans and precise health management schemes. This consequently enabled adjustment of treatment schemes in real time, reduced the number of admissions due to acute exacerbations, and lowered the readmission rate. For hospitals interested in implementing a similar model, we suggest first conducting a detailed review of the current situation prior to making adequate changes based on the relevant disease and patient populations.

Consequently, the HIS-based case information management model could improve efficiency, enhance the quality of case management, and aid in stabilizing the conditions of patients with chronic respiratory diseases. In contrast to the hospital case management information system reported by Yuan et al [22], the system described in this study includes a personal terminal app. Previous studies confirmed that a stand-alone mobile health app could improve patient compliance and disease control [6-8]; thus, whether this system can be used to manage specialized disease cohorts for patients with chronic diseases remains to be determined. In this study, the effect on the retention rate of patients was confirmed; however, the overall operational indicators for the diagnosis and treatment of chronic diseases should be further determined.

### Conclusion

With the advancement of information technology, the internet and medical technology have been applied to the management of chronic diseases. As an information-based platform for the case management of patients with chronic respiratory diseases, a newly developed chronic disease management information system was introduced in this study. This system is capable of designing the follow-up time registration, follow-up content, approaches, methods, quality control, and feedback process for a single chronic respiratory disease via the single-disease clinical pathway following the case management process (enrollment, assessment, planning, implementation, feedback, and evaluation). Use of this system can encourage patients with chronic respiratory diseases to adhere to regular follow-up and form an outpatient-inpatient-home chronic disease management strategy. This can help in reducing the admission rate for acute exacerbations, increase the return visit rate, and improve the correctness and compliance of home self-management of patients with chronic respiratory diseases. Owing to these benefits, wide adoption of such information systems for the management of chronic diseases can offer substantial economic and social value.

## Abbreviations

COPD: chronic obstructive pulmonary disease
HIS: hospital information system
*ICD*: *International Classification of Diseases*
